# Supplementary material for: Examination of the spatial-temporal variations in terrestrial water reserves and green efficiency of water resources in China’s three northeastern provinces
Source: PLoS One. 2025 Aug 6;20(8):e0328674. doi: 10.1371/journal.pone.0328674 (PMC12327649; doi:10.1371/journal.pone.0328674)
Supplement: Attachment — Submitted filename: Response to Reviewers.docx [file pone.0328674.s001.docx]

Dear Reviewer and Editor:

We sincerely appreciate the constructive comments provided by the reviewers and editors regarding our manuscript. Your insights and guidance have served as a ladder for our continuous improvement, significantly enhancing the quality of our work. We deeply regret the issues identified in the manuscript and assure you that we will internalize the advice from experts in our future research and studies, striving for higher levels of scholarly achievement. Below is a detailed response addressing each comment. The reviewers' comments are referenced using italicized font and numbering, while our responses are presented in regular font. Modifications and additions are highlighted in red for emphasis.

***Recommendation 1****:*  *Please ensure that your manuscript meets PLOS ONE's style requirements, including those for file naming.*

**Response 1:**

Thank you very much for your suggestion. I have made the modifications according to PLOS ONE's style requirements

***Recommendation 2****:*  *In your Methods section, please include additional information about your dataset and ensure that you have included a statement specifying whether the collection and analysis method complied with the terms and conditions for the source of the data.*

**Response 2:**

Thank you very much for your suggestion. The data is publicly available. And the data collection and analysis method complied with the terms and conditions set by the University of Texas for the use of these data

***Recommendation 3****:* *Please amend either the title on the online submission form (via Edit Submission) or the title in the manuscript so that they are identical.*

**Response 3:**

Thank you very much for your suggestion. The modifications have been made as required.

***Recommendation 4****:* *We note that Figures 1 and 2 in your submission contain [map/satellite] images which may be copyrighted. All PLOS content is published under the Creative Commons Attribution License (CC BY 4.0), which means that the manuscript, images, and Supporting Information files will be freely available online, and any third party is permitted to access, download, copy, distribute, and use these materials in any way, even commercially, with proper attribution. For these reasons, we cannot publish previously copyrighted maps or satellite images created using proprietary data, such as Google software (Google Maps, Street View, and Earth).*

**Response 4:**

Thank you very much for your suggestion. I have replaced the image with copyright issues.

***Recommendation 5****:* *This articel is designed to evaluate the spatial-temporal variations in terrestrial water reserves and the efficiency of green water usage in China's three northeastern provinces. The overall methodology and procedures were valid. However, the ABSTRACT should be re-summarized as there are no summarized conclusions and proper implications.*

**Response 5:**

Thank you very much for your suggestions and advice. It is your help that enables me to continually improve. It is your guidance that has enabled us to continually improve. I have rewritten the abstract and summarized the conclusion again.

***Recommendation 6****:* *The entire paper should use a two end aligned format.*

**Response 6:**

Thank you very much for your suggestions and advice. It is your help that enables me to continually improve. It is your guidance that has enabled us to continually improve. The article has been revised to align both ends.

***Recommendation 7****:* *The title level is chaotic, for example, 1, 2, 3, etc. appear below 2.2.1.*

**Response 7:**

Thank you very much for your suggestions and advice. It is your help that enables me to continually improve. It is your guidance that has propelled our continuous improvement. The title number of the article has been modified.

***Recommendation 8****:* *The introduction section should elaborate on the importance and significance of this research in detail.*

**Response 8:**

Thank you very much for your suggestions and advice. It is your help that enables me to continually improve. The introduction section of the article has been rewritten to supplement and elaborate on the importance and significance of the research.

***Recommendation 9****:* *The first column of Table 2, Table 4 have two Year headers that need to be modified.*

**Response 9:**

Thank you very much for your suggestions and advice. The corresponding issues have been modified.

***Recommendation 10****:* *The clarity of most figures is low and needs to be improved.*

**Response 10:**

Thank you very much for your suggestions and advice. The corresponding issues have been modified.

***Recommendation 11****:* *The paper mentions the use of Tobit model to analyze the influencing factors of water resource utilization efficiency, but the application and analysis of formulas (7) and (8) in Tobit model are not used in the paper.*

**Response 11:**

Thank you very much for your suggestions and advice. The article has added a fifth part, which supplements the application and analysis of Tobit model.

***Recommendation 12****:* *In the analysis of innovative points in the research content, there is no explanation of the innovative content, but rather a more accurate description of the research content is needed.*

**Response 12:**

Thank you very much for your suggestions and advice. The article has supplemented the innovation in the abstract and introduction sections.

***Recommendation 13****:* *What is the basis for determining the evaluation index system?*

**Response 13:**

Thank you very much for your suggestions and advice. In section 2.2.3 of the article, the selection criteria for indicators have been supplemented.

***Recommendation 14****:* *I highly recommend to write limitations of the study and recommendation for future study at the end of conclusion.*

**Response 14:**

Thank you very much for your suggestions and advice. The conclusion section of the paper supplements the limitations of the research and the next steps of research.

***Recommendation 15****:* *The discussion on policies is not in-depth enough. It is recommended to include more policy analysis.*

**Response 15:**

Thank you very much for your suggestions and advice. In the introduction section, an analysis and discussion of policies have been added
